# Generation of monoclonal antibodies against MGA and comparison of their application in breast cancer detection by immunohistochemistry

**DOI:** 10.1038/srep13073

**Published:** 2015-08-14

**Authors:** Cuimi Duan, Xiqin Yang, Xuhui Zhang, Jiannan Feng, Zhiqiang Liu, Haiping Que, Heather Johnson, Yanfeng Zhao, Yawen Fan, Yinglin Lu, Heqiu Zhang, Yan Huang, Bingshui Xiu, Xiaoyan Feng

**Affiliations:** 1 Department of Bio-diagnosis, Institute of Basic Medical Sciences, 27, Taiping Road, Beijing, 100850, China; 2 Department of Immunology, Institute of Basic Medical Sciences, 27, Taiping Road, Beijing, 100850, China; 3 Olympia Diagnostics, Inc. Sunnyvale, CA 94086, USA; 4 Affiliated 307 Hospital, Beijing, 100071, China

## Abstract

Mammaglobin A (MGA) is an organ specific molecular biomarker for metastatic breast cancer diagnosis. However, there are still needs to develop optimal monoclonal antibodies (mAbs) to detect MGA expression in breast carcinoma by immunohistochemistry. In this study, we first generated mAbs against MGA. Then, we used epitope prediction and computer-assisted structural analysis to screen five dominant epitopes and identified mAbs against five epitopes. Further immunohistochemical analysis on 42 breast carcinoma specimens showed that MHG1152 and MGD785 had intensive staining mainly in membrane, while CHH11617, CHH995 and MJF656 had more intensive staining within the cytoplasm. MGA scoring results showed that MJF656 had the highest rate (92.8%) of positive staining among five mAbs, including higher staining intensity when compared with that of MHG1152 (p < 0.01) and CHH995 (p < 0.05) and the highest the mean percentage of cells stained among mAbs. Furthermore, we analyzed the relationship of positive staining rate by mAbs with patient clinical characteristics. The results suggest that MJF656 was able to detect MGA expression, especially in early clinical stage, low grade and lymph node metastasis-negative breast carcinoma. In conclusion, our study generated five mAbs against MGA and identified the best candidate for detection of MGA expression in breast cancer tissues.

Breast cancer is the most prevalent cancer in women and the second leading cause of cancer-related death in women worldwide^1^. The incidence and mortality of breast cancer continue to rise, not only in the western world^2^, but also in Asian countries^3^. Distant site metastases of breast cancer is the main cause of death, thus improvement in early detection and diagnosis of breast cancer metastasis will contribute to reduction of breast cancer mortality.

Mammaglobin A (MGA) is a membrane-associated 93-amino acid protein that belongs to the secretoglobin superfamily^4^,^5^. It has been shown that MGA expression is limited to breast organ and it is expressed at a lower level in normal breast epithelium, but at a higher level in breast cancer tissue^6^. Importantly, MGA positive or high level expression by immunohistochemical staining was found in approximately 80 ~ 90% of intraductal carcinoma and invasive ductal carcinoma^7^. MGA has been used as a serum biomarker for breast cancer diagnosis and prognosis^6^,^8^,^9^,^10^,^11^,^12^,^13^. Using the nested reverse transcriptase polymerase chain reaction (RT-PCR) assay, MGA could be more easily detected in the metastatic breast cancer group than the healthy controls and breast cancer without metastasis group in the peripheral blood samples^14^. The commonly used breast cancer biomarkers including carcinoembryonic antigen (CEA) and CA15-3 are rarely elevated at early metastatic stage and are not elevated in many patients with metastases^15^,^16^. Because of its specific and differential expression in the mammary tissue, MGA may serve as a breast cancer-specific biomarker for evaluating secondary tumors from unknown primary sites^17^,^18^,^19^,^20^,^21^,^22^. More importantly, MGA may be used as a metastatic breast cancer biomarker to detect the presence of micrometastasis in the bone marrow^23^ and lymph node^24^. The sensitivity and specificity of detection of breast cancer lymph node metastases can be reached at 90% and 94%, respectively when MGA was combined with cytokeratin-19 (CK19) and used as a diagnostic test^24^. Thus, MGA has been used as a specific biomarker for diagnosis of breast cancer metastasis with immunohistochemical method^18^,^19^,^25^,^26^.

However, present commercially available MGA mAbs for immunohistochemical staining showed limited sensitivity and specificity. In light of the importance of MGA in breast cancer diagnosis and prognosis as reported above, it is urgent to generate effective antibodies for specific detection of MGA with good immunohistochemical reactivity in breast carcinoma tissues. In this study, we generated several MGA mAbs after performing epitope prediction coupled with computational modeling and docking analysis. The characteristics of mAbs generated was evaluated and compared for detection of MGA expression by immunohistochemistry. In addition to development of a MGA mAb with good immunohistochemical reactivity, our study revealed that epitope prediction followed by computational modeling and docking analysis is a good strategy for generation of mAbs.

## Results

### MAbs Generation and Epitopes prediction of MGA

**Figure 1:**
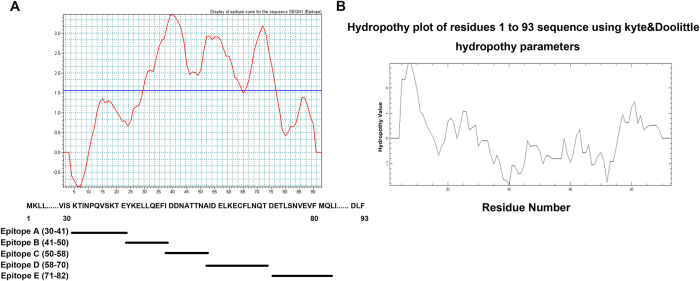
Epitope prediction and hydrophobic analysis of MGA. (**A**) Five antigen dominant epitopes of MGA were predicted as shown. Below the graph, the sequences of the synthetic peptides used for identification were shown. (**B**) The hydrophobic and hydrophilic properties analysis of different regions of MGA.

Generation of mAbs was conducted as shown in Materials and Methods. For selection and identification of mAbs, we first used Biosun software to predict dominant epitopes of MGA protein. As shown in Fig. 1A, five dominant epitopes (A–E) were predicted, the relative sequences of which are shown below the graph. Using the Kyte & Doolittle hydropathy method, the hydrophobic and hydrophilic properties of MGA were studied. As shown in Fig. 1B, the four epitopes (epitope A-D) all possessed strong hydrophobic region while the last epitope, epitope E, had a hydrophilic region at 71 to 82 amino acid residues.

### 3-D structure modeling and theoretical prediction of the physical-chemical property of MGA

**Figure 2:**
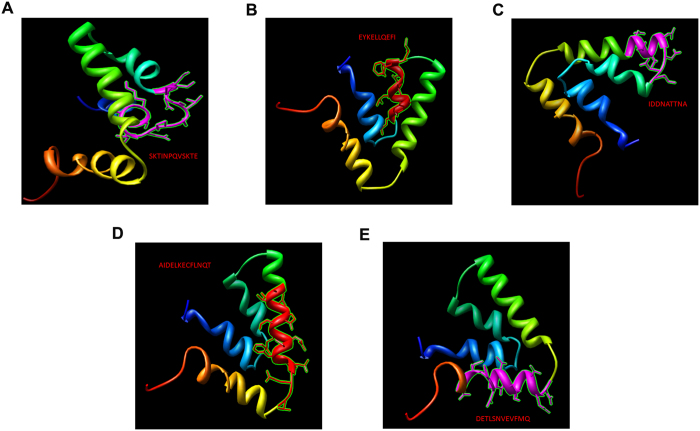
MGA structure modeling. Ribbon representation denoted the 3-D theoretical structure of MGA derived from computer-guided modeling. Five epitope regions with side chains highlighted in colors were shown in A–E. (**A**) epitope A was highlighted in purple. (**B**) epitope B was highlighted in red. (**C**) epitope C was highlighted in purple. (**D**) epitope D was highlighted in red. (**E**) epitope E was highlighted in purple.

To identify whether the above five epitope regions of MGA are involved in antigen-antibody interactions, the stable 3-D structure of MGA was constructed using computer-guided homology modeling method as shown in Fig. 2. In addition, the epitopes mentioned above were displayed in the 3-D structure. As shown in Fig. 2, all the epitopes were located in the turn region of the 3-D structure of MGA. The core amino acid residues were exposed to the solvent and can interact with the screened antibody easily. Therefore the above five epitopes are good candidates for mAbs selection and identification. Meanwhile, the antibody isotype was identified on each antibodies generated (Supplementary Table 1).

### Validation of MGA mAbs by immunohistochemical staining

**Figure 3:**
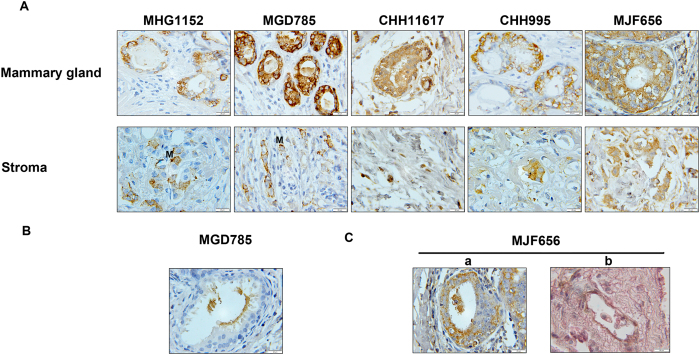
Pattern of staining by MGA mAbs in breast carcinoma tissues. (**A**) Representative images showing immnuostaining of MHG1152, MGD785, CHH11617, CHH995 and MJF656A in breast cancer mammary gland and stroma. (**B**) Representative images showing staining of MGD785 in the secretions of mammary gland. (**C**) Representative images showing staining of MJF656 in tumor cells that shed into the mammary gland cavity (a) and blood vessel (b). a: MGA staining, b: MGA and CD34 double staining (MGA: pink, CD34: brown). Formalin-fixed, paraffin-embedded sections of breast carcinoma were stained with the indicated MGA mAbs. Scale bar: 20 μm, magnification: ×100. “M” indicates MGA staining on the cell membrane.

To investigate the reactivity of the generated mAbs in the detection of MGA on serial paraffin-embedded breast carcinoma tissue sections, immunohistochemistry was performed on 42 breast carcinoma samples. Clinical and Pathological Characteristics of Breast Cancer Patients were shown in Table 1. As shown in Fig. 3A, anti-MGA immunostaining of MHG1152 and MGD785 mAbs was patchy and occurred in both cytoplasm and cell membrane, mainly in cell membrane, whereas anti-MGA immunostaining of CHH11617, CHH995 and MJF656 was more extensive within the cytoplasm (Fig. 3A).

As shown in Fig. 3B, MGD785 showed clear staining in the secretions of the mammary gland, which were not found by other mAbs. Importantly, staining with MJF656 was seen in tumor cells that shed into both the mammary gland cavity and the blood vessels (Fig. 3C). The above data showed that MGA mAbs against different epitopes have different immunostaining patterns in breast cancer tissues.

### Characterization of mAbs according to positive staining rate

**Figure 4:**
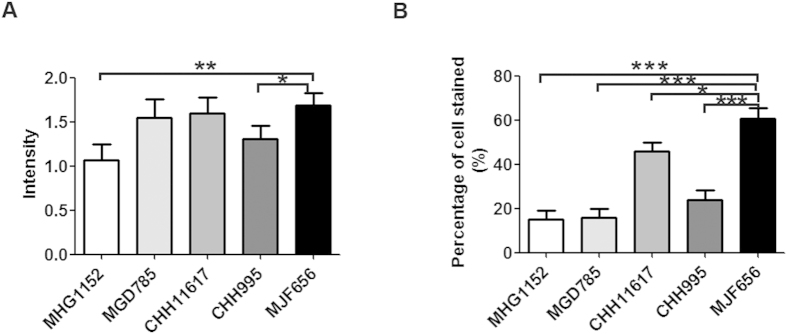
Comparison of immunohistochemical staining results of mAbs measured by staining intensity and percentage of cells stained. (**A**) The immunohistochemical staining intensity of mAbs. (**B**) The percentage of cells stained by mAbs. *p < 0.05, **p < 0.01, ***p < 0.001. Unpaired t test with Welch’s correction was used to compare the results of staining intensity or percentage of cells stained of MJF656 with other mAbs. We analyzed three sections of each breast cancer tissue for each MGA mAb.

To further characterize reactivity of the mAbs in immunostaining, the positive staining rate was scored according to the staining intensity and percentage of stained cells. As shown in Supplementary Table 2, unequivocal positive staining was seen in 39 of 42 cases (92.8%) with MJF656 Ab, whereas positive staining was only observed in 22 of 42 cases (52.4%) with MHG1152 Ab. 16 of 42 cases had scored as +3 when stained with MGD785 Ab and the positive rate is 57.1%. To better evaluate the mAbs, the staining intensity and percentage of cells stained by each mAb were plotted and compared as shown in Fig. 4. The difference in intensity between MJF656 and MHG1152 was statistically significant (p < 0.01), and the difference between MJF656 and CHH995 is also significant (p < 0.05) (Fig. 4A). However, no significant difference was found when comparing the staining intensity of MJF656 with that of MGD785, or CHH11617 (Fig. 4A). As shown in Fig. 4B, the mean percentage of stained cells with MJF656 was the highest among all five mAbs. The above data suggests that MJF 656 is the best of the five mAbs for MGA immunostaining in breast carcinoma tissues.

**Figure 5:**
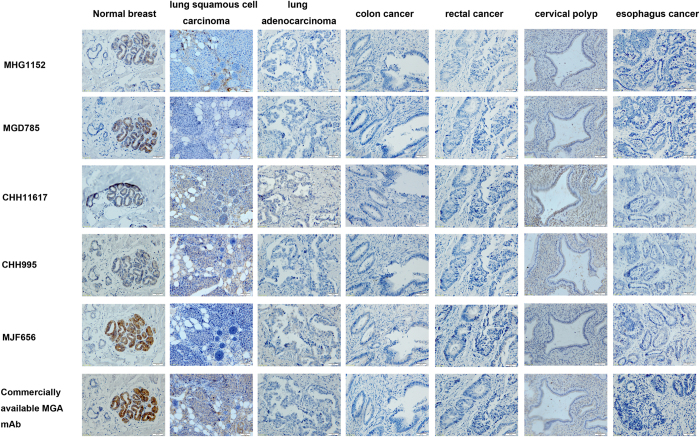
Representative immunohistochemical staining of MGA mAbs in non-breast tumors and tissues. Normal breast tissues were done as positive control. Scale bar: 50 μm, Magnification, ×200.

The sensitivity and specificity of MGA mAbs was summarized in Table 2. The sensitivity of mAbs was assessed by the positive expression of the MGA in 42 cases breast carcinoma. It was clear that MJF656 has a higher diagnostic sensitivity compared with other MGA mAbs, p value is <0.001 in all cases except for comparison with CHH11617. The specificity of MGA mAbs were assessed the expression in lung squamous cell carcinoma, lung adenocarcinoma, colon cancer, rectal cancer, cervical polyp and esophagus cancer which are supposed to have no MGA protein expression. But the staining with MHG1152, MGD785, CHH11617, CHH995 and commercially available MGA mAb were seen in lung squamous cell carcinoma and cervical polyp (Fig. 5). No staining with any of the tested MGA mAbs was seen in lung adenocarcinoma, colon cancer, rectal cancer and esophagus cancer (Fig. 5). MJF656 showed specific staining with normal breast tissue (Fig. 5).

### Evaluation of clinical characteristics of cancer cases with positive expression of mAbs

**Figure 6:**
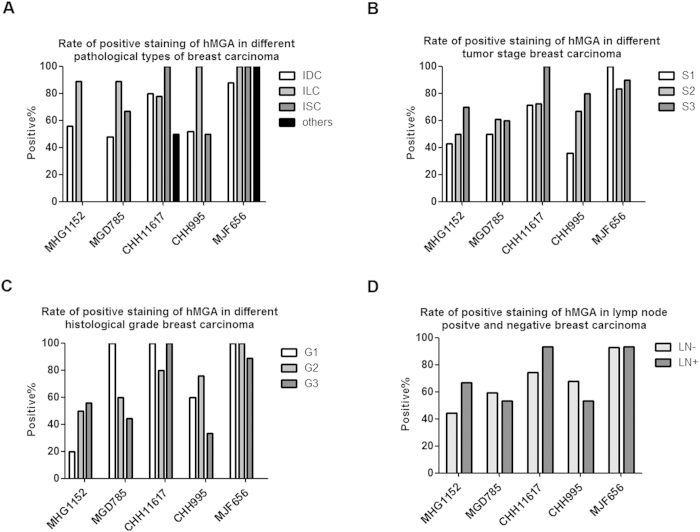
Relationship of the rate of positive mAb staining with patient clinical characteristics. (**A–D**) Relationships of the rate of positive mAb staining with patients’ pathological type, tumor stage, histological grade and lymph node metastasis. IDC: infiltrating ductal carcinoma, ILC: infiltrating lobular carcinoma, ISC *in situ* carcinoma, LN-: lymp node metastasis negative, LN+: lymp node metastasis positive.

The relationship of Ab reactivity in immunostaining with clinical characteristics of the 42 cases was analyzed based on patient clinical data including breast cancer pathological type, histological grade, clinical stage and lymph node metastasis (Fig. 6). Statistical analysis results were shown in Supplementary Table 3. Figure 6A showed positive MGA expressions in different breast cancer pathological types. MJF656 had the significantly highest positive staining rate (88%) in the infiltrating ductal carcinoma (IDC) as compared with the other antibodies (p < 0.05 in all cases, except for CHH11617). The infiltrating lobular carcinoma (ILS) and *in situ* carcinoma (ISC) stained with MJF656were positive in 100% of cases. Furthermore, CHH995 showed positive staining in all of the 9 ILS, yet very low reactivity with the other two breast cancer pathological types. As shown in Fig. 6B, all of the 14 cases of Stage 1 breast carcinoma showed staining with MJF656; p value is less than 0.01 in all cases as compared with MGD785, CHH995 and MHG1152. As to Stage 2 breast carcinoma, the positive staining rate of MJF656 is higher than MHG1152 (p < 0.05) (Supplementary Table 3). When comparing the MGA positive expression according to histological grade, MGD785, CHH11617 and MJF656 showed good staining in all 5 G1 tissue samples (Fig. 6C). The reactivity of MJF656 in G1 breast carcinoma is better than MHG1152 (p = 0.05) (Supplementary Table 3). Importantly, all of the lymph node metastasis-negative breast cancer samples were stained with MJF656 (Fig. 6D). The positive staining rate of MJF656 in lymph node metastasis-negative breast cancer samples is significantly higher than that of MGD785, CHH995 or MHG1152 (p < 0.01 in all cases) (Supplementary Table 3). Overall, MJF656 might be the best candidate for MGA detection in early stage, low clinical grade and lymph node metastasis-negative breast cancer patients. Whereas CHH11617 may be more appropriate for late stage, high grade and lymph node metastasis-positive breast cancer cases as compared with MGD785 or CHH995 (Fig. 6) (Supplementary Table 3).

## Discussion

As a breast tumor specific biomarker, MGA is an important tool for detecting primary and metastatic breast cancer, and monitoring breast cancer lymph node metastasis with immunohistochemical method. Therefore, we set out to develop specific mAbs to detect the expression of MGA in breast tumor tissue by immunohistochemical analysis. There is no reliable method to predict which mAb will work in immunohistochemistry, thus we performed immunohistochemical staining on serial paraffin embedded breast carcinoma sections using the five generated mAbs. We found that all of the five mAbs generated work well in immunohistochemical staining, although the staining intensity and percentage of cells stained with the mAbs were different. Among them, MJF656 had the highest positive staining rate of 92.8%. Further, we studied the relationships of the staining of the mAbs with clinical characteristics of breast cancer patients. These studies showed that MJF656 is the most appropriate mAb for detecting MGA protein by immunohistochemistry in early stage, low grade breast carcinoma. This could be due, at least in part, to the higher affinity of MJF656 toward the epitope used to generate the Ab as compared with other mAbs.

In this study, we also found membrane staining of MGA using MHG1152 and MGD785, but not with the other three mAbs. The hydrophobic analysis showed that peptide A–D regions against which MHG1152, MGD785, CHH11617 and CHH995 were generated are hydrophobic, while peptide E region against which MJF656 was generated is hydrophilic, which might give good explanations for the best cytoplasm staining by MJF656. Furthermore, the difference in immunostaining by different mAbs might also be due to the exposure of antigen epitope in different environment (such as blood, mammary secretions) is different. Moreover, the membrane-associated MGA protein may be utilized for breast cancer targeted drug delivery, thus MHG1152 and MGD785 might be used as target antibody for future MGA targeted therapy. Because the specific staining for the exfoliated cells, MJF656 might be used for detection of breast cancer erosion into the blood vessels in ice section during surgery. In particular, MJF656 might be a good candidate antibody for identification of breast cancer origin cells that was captured by CTC detection system (CELLSEARCH® Circulating Tumor Cell (CTC) Test) as specific staining in the exfoliated cells. In addition, our study on application of the five mAbs in the detection of MGA in the sera is already underway.

mAbs generated against different epitopes showed different immunohistochemisty reactivity and staining pattern, which revealed the choice of antibody is a key factor that could affect reproducibility and quantitative value of immunohistochemical staining. In addition, improper antibody choice will result in incorrect clinical prediction results. Importantly, the present study proved that computer-assisted protein structural analysis is a useful tool for development of diagnostic reagents for antigen detection in laboratory and clinical samples.

## Methods

### Immunization of mice and generation of MAbs

All experimental protocols related to animal work were approved by the Animal Care Committee, Institute of Basic Medical Sciences. All methods were carried out in accordance with the approved guidelines of the Animal Care Committee. pBV220/hMGA (8-93aa) was used as an immunogen to six-week-old female BALB/c mice. The animals were immunized three times subcutaneously, at approximately 4 week intervals, with 50 μg of the protein in complete Freund’s adjuvant (Sigma). Three days after the last injection, spleen cells from immunized mice were fused with Sp2/0 myeloma cells (ATCC) at a ratio of 10: 1. Hybridomas were selected and positive wells were identified using indirect ELISA with MGA peptides A–E (see Fig. 1A) as antigens. mAbs were purified with Melon™ Gel Monoclonal IgG Purification Kit (Thermo scientific) and was isotyped with Rapid Isotyping Kit (Thermo scientific).

### Patient Data

All the patients were properly informed, and written informed consent was obtained from each individual. All methods were carried out in accordance the approved guidelines of Ethics Committee of Institute of Basic Medical Sciences and Affiliated 307 Hospital. 42 patients diagnosed with breast cancer, aged between 29 and 86 years old, were enrolled in the study. Their primary tumors were surgically removed and subjected to immunohistochemical analysis. Characterization of the patients was based on their tumor stage and lymph node metastasis, which is shown in Table 1. Among the 42 breast cancer patients investigated, the most frequently occurring cancer was invasive ductal carcinoma (59.5%), followed by infiltrating lobular carcinoma (21.4%). 61.9% of the patients were negative for lymph node metastasis. The most frequently occurring clinical stage was stage II (42.9%) and 59.5% of the patients were in histological grade II.

### Immunohistochemical Analysis

Briefly, 3 μm sections were obtained from formalin-fixed, paraffin-embedded (PPFE) cell block preparations. Heat-induced epitope retrieval was applied with 0.02 M concentration of citrate buffer (pH 6.0) in a heater for 10 min. Immunostaining was performed with mAbs against MGA at 4 °C overnight in a dark humid chamber. After washing the slides in PBS, a streptavidin-biotin system was applied. DAB (3, 3′-diaminobenzidine) was used for color development. The sections were counterstained with hematoxylin, dehydrated and mounted with neutral gum. Appropriate positive and negative control slides were prepared.

For MGA and CD34 double staining, the sections were incubated with a 1:1 volume mixture of mouse mAb(MJF656) and Rabbit CD34 mAb(ZA0550, ZSGB-BIO, Beijing) in a buffer (1% bovine serum albumin in PBS) at 4 °C overnight in a dark humid chamber. Sections were firstly incubated with HRP conjugated goat anti-rabbit antibody (ZB2301, ZSGB-BIO, Beijing) for 0.5 h at room temperature and DAB were used for color development. Then, the sections were incubated with AP conjugated goat anti-mouse secondary antibody (SAP-9102, ZSGB-BIO, Beijing) followed by color development with AP-red (ZLI-9042, ZSGB-BIO, Beijing). Finally, sections were counterstained with hematoxylin, dehydrated and mounted with Clearmount (ZLI9553, ZSGB-BIO, Beijing).

### Computational modeling and minimizations

Based on the sequence of MGA deposited in Swissport database (code ID, Q13296), the secondary structure of MGA was analyzed using GOR IV method^27^,^28^. Using computer-guided homology modeling methods^27^ the 3-D structure of MGA was constructed on the basis of the predicted secondary structure of MGA. With the modeled 3-D structure of MGA as the initial conformation, the optimized 3-D structure of MGA was obtained based on molecular minimization method under CVFF and Charmm force field^27^. All calculations were performed using InsightII2000 software (MSI, San Diego, CA) on SGI workstation (www.sgi.com).

### Mammaglobin Scoring

The scoring of MGA in the tissue samples was based on staining intensity and percentage of stained cell as the following: negative (0), weak positive with less than 10% stained cells (+1), moderate positive with 11 to 50% stained cells (+2), and strong positive with over 51% stained cells (+3). The scoring of MGA is shown in Supplementary Table 2. The score >+1 was regarded as Positive staining.

### Statistical Analysis

The staining intensity and percentage of cells stained with each antibody were compared by using unpaired t test with Welch’s correction. Pearson chi-square and Fisher’s exact test were used for comparisons in sensitivity and specificity comparisons and the rate of positive mAb staining with patient clinical characteristics comparisons of MGA mAbs. In all cases, p < 0.05 was considered statistically significant.

## Additional Information

**How to cite this article**: Duan, C. *et al.* Generation of monoclonal antibodies against MGA and comparison of their application in breast cancer detection by immunohistochemistry. *Sci. Rep.* **5**, 13073; doi: 10.1038/srep13073 (2015).

## Supplementary Material

Supplementary Information

## Figures and Tables

**Table 1:** Clinical and Pathological Characteristics of Breast Cancer Patients used for Immunohistochemical Staining of MGA.

**Characteristic**	**n (%)**
Age (years)	
<50	19 (45.2)
50–60	16 (38.1)
>60	7 (16.7)
Histological type	
Infiltrating ductal carcinoma	25 (59.5)
Infiltrating lobular carcinoma	9 (21.4)
*In situ* carcinoma	6 (14.3)
Others	2 (4.8)
Clinical stage	
S1	14 (33.3)
S2	18 (42.9)
S3	10 (23.8)
Lymph node metastasis	
N−	26 (61.9)
N+	15 (35.7)
NA	1 (2.4)
Histological grading	
G1	5 (12.0)
G2	25 (59.5)
G3	9 (21.4)
NA	3 (7.1)

NA: data not available.

**Table 2:** Diagnostic sensitivity and specificity of MGA mAbs evaluated in this study in non-breast tissues and tumors.

**Antibody**	DiagnosticSensitivity	p value(vs MJF656)	DiagnosticSpecificity	p value(vs MJF656)
MHG1152	52.4%	0.000	67.7%	0.000
MGD785	57.1%	0.000	67.7%	0.000
CHH11617	81.0%	0.106	67.7%	0.000
CHH995	59.5%	0.000	67.7%	0.000
MJF656	92.8%		100%	
Commercially available MGA mAb	57.1%	0.004	67.7%	0.000
