# Supplementary material for: Generation of monoclonal antibodies against MGA and comparison of their application in breast cancer detection by immunohistochemistry
Source: Sci Rep. 2015 Aug 14;5:13073. doi: 10.1038/srep13073 (PMC4536492; doi:10.1038/srep13073)
Supplement: Supplementary Information [file srep13073-s1.pdf]

Supplementary Figures and Tables

Generation of monoclonal antibodies against MGA and comparison of their application in breast cancer detection by immunohistochemistry

Cuimi Duan, Xiqin Yang, Xuhui Zhang, Jiannan Feng, Zhiqiang Liu, Haiping Que, Heather Johnson, Yanfeng Zhao, Yawen Fan, Yinglin Lu, Heqiu Zhang, Yan Huang, Bingshui Xiu, XiaoyanFeng

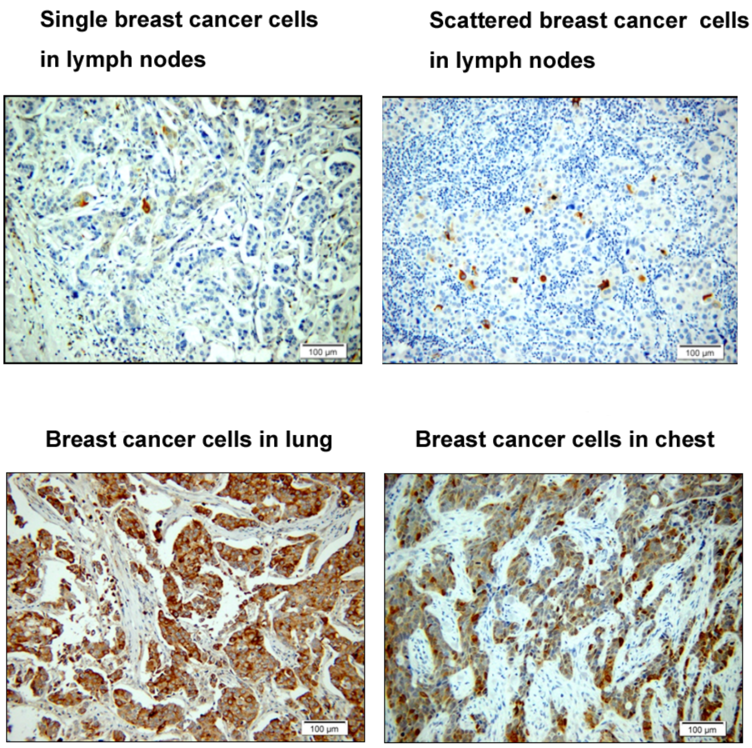

Supplementary Figure 1.MGA specific stainings in breast cancer cells.
MGA mAbs (MJF656) had specific staining in breast cancer cells that metastasized to lymph nodes, lung and chest wall. Scale bar: 100 μm, magnification: ×200.

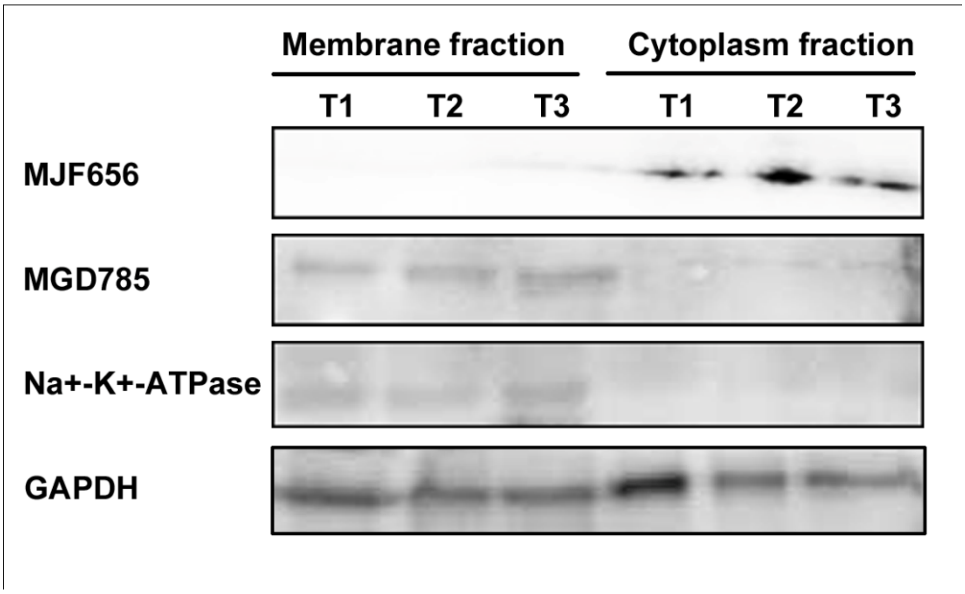

Supplementary Figure2. Western blot analysis of MGA protein in both membranous and cytoplasmic proteins of breast cancer tissue with MJF656 and MGD785. GAPDH was used as loading control.

Supplementary Table1 Isotypes of MGA mAbs

**Epitope**	**Monoclonal Antibody**	**Isotype**
A	MHG1152	IgG2b
B	MGD785	IgG2a
C	CHH11617	IgG2a
D	CHH995	IgG2a
E	MJF656	IgG1

Supplementary Table2 MGA mAb Staining Scores in Breast Cancer Patient Specimens

**Score**	**n**	**0**	**+1**	**+2**	**+3**	**Positive rate**
MHG1152	42	20	9	9	4	52.4%
MGD785	42	18	1	7	16	57.1%
CHH11617	42	8	11	18	5	81.0%
CHH995	42	17	1	12	12	59.5%
MJF656	42	4	13	19	7	92.8%
Commercially available MGA	42	18	6	11	7	57.1%

Supplementary Table3. p value of comparison of mAb stainings for evaluation of clinical characteristics of cancer cases.

The mAb stainings for evaluation of clinical characteristics of cancer cases as shown in Figure. 6 were compared and p values were calculated using Pearson chi-square or Fisher's exact probability test. The choice of statistical method was based on the following rules: Pearson Chi-square was used when T>5 and N>40, continuity correction Chi-square was used when 1<T<5 and N>40, Fisher's exact probability test was used when T<1 and N≤40. T: minimum expected count, N: total number of observations.

Group		Antibody comparison	p value
**Pathological type**	**IDC**	MJF656 vs Commercially available MGA mAb	0.047
		MJF656 vs MGD785	0.002
		MJF656 vs CHH995	0.005
		MJF656 vs MHG1152	0.012
		MJF656 vs CHH11617	0.700
	**ILC**	MJF656 vs Commercially available MGA mAb	1.000
		MJF656 vs MGD785	1.000
		MJF656 vs MHG1152	1.000
		MJF656 vs CHH11617	0.453
	**ISC**	MJF656 vs Commercially available MGA mAb	0.182
		MJF656 vs MGD785	0.439
		MJF656 vs CHH995	0.182
		MJF656 vs MHG1152	0.004
	**other**	MJF656 vs Commercially available MGA mAb	0.019
		MJF656 vs MGD785	0.019
		MJF656 vs CHH995	0.019
		MJF656 vs MHG1152	0.019
		MJF656 vs CHH11617	0.189
**Tumor stage**	**S1**	MJF656 vs Commercially available MGA mAb	0.009
		MJF656 vs MGD785	0.009
		MJF656 vs CHH995	0.001
		MJF656 vs MHG1152	0.003
		MJF656 vs CHH11617	0.105
	**S2**	MJF656 vs Commercially available MGA mAb	0.07
		MJF656 vs MGD785	0.246
		MJF656 vs CHH995	0.441
		MJF656 vs MHG1152	0.034
		MJF656 vs CHH11617	0.688
	**S3**	MJF656 vs Commercially available MGA mAb	0.576
		MJF656 vs MGD785	0.302
		MJF656 vs CHH995	1.000
		MJF656 vs MHG1152	0.576

		MJF656 vs CHH11617	1.000
**Histological grade**	**G1**	MJF656 vs Commercially available MGA mAb	0.429
		MJF656 vs CHH995	0.429
		MJF656 vs MHG1152	0.05
	**G2**	MJF656 vs Commercially available MGA mAb	0.000
		MJF656 vs MGD785	0.000
		MJF656 vs CHH995	0.03
		MJF656 vs MHG1152	0.003
		MJF656 vs CHH11617	0.059
	**G3**	MJF656 vs Commercially available MGA mAb	0.293
		MJF656 vs MGD785	0.134
		MJF656 vs CHH995	0.053
		MJF656 vs MHG1152	0.293
		MJF656 vs CHH11617	0.317
**Lymph node metastasis**	**LN+**	MJF656 vs Commercially available MGA mAb	0.084
		MJF656 vs MGD785	0.039
		MJF656 vs CHH995	0.039
		MJF656 vs MHG1152	0.177
		MJF656 vs CHH11617	1.000
	**LN-**	MJF656 vs Commercially available MGA mAb	0.002
		MJF656 vs MGD785	0.004
		MJF656 vs CHH995	0.009
		MJF656 vs MHG1152	0.000
		MJF656 vs CHH11617	0.144
**Pathological type**	**IDC**	CHH11617 vs Commercially available MGA mAb	0.208
		CHH11617 vs MGD785	0.018
		CHH11617 vs CHH995	0.037
		CHH11617 vs MHG1152	0.069
	**ILC**	CHH11617 vs Commercially available MGA mAb	1.000
		CHH11617 vs MGD785	1.000
		CHH11617 vs CHH995	0.453
		CHH11617 vs MHG1152	1.000
	**ISC**	CHH11617 vs Commercially available MGA mAb	0.782
		CHH11617 vs MGD785	0.439
		CHH11617 vs CHH995	0.182
		CHH11617 vs MHG1152	0.004
	**other**	CHH11617 vs Commercially available MGA mAb	1.000
		CHH11617 vs MGD785	1.000
		CHH11617 vs CHH995	1.000
		CHH11617 vs MHG1152	1.000
**Tumor stage**	**S1**	CHH11617 vs Commercially available MGA mAb	0.246
		CHH11617 vs MGD785	0.246
		CHH11617 vs CHH995	0.246

		CHH11617 vs MHG1152	0.058
	**S2**	CHH11617 vs Commercially available MGA mAb	0.298
		CHH11617 vs MGD785	0.480
		CHH11617 vs CHH995	0.717
		CHH11617 vs MHG1152	0.171
	**S3**	CHH11617 vs Commercially available MGA mAb	0.210
		CHH11617 vs MGD785	0.094
		CHH11617 vs CHH995	0.456
		CHH11617 vs MHG1152	0.210
**Histological grade**	**G1**	CHH11617 vs Commercially available MGA mAb	0.429
		CHH11617 vs CHH995	0.429
		CHH11617 vs MHG1152	0.05
	**G2**	CHH11617 vs Commercially available MGA mAb	0.123
		CHH11617 vs MGD785	0.123
		CHH11617 vs CHH995	0.733
		CHH11617 vs MHG1152	0.208
	**G3**	CHH11617 vs Commercially available MGA mAb	0.089
		CHH11617 vs MGD785	0.035
		CHH11617 vs CHH995	0.012
		CHH11617 vs MHG1152	0.089
**Lymph node metastasis**	**LN+**	CHH11617 vs Commercially available MGA mAb	0.084
		CHH11617 vs MGD785	0.039
		CHH11617 vs CHH995	0.039
		CHH11617 vs MHG1152	0.171
	**LN-**	CHH11617 vs Commercially available MGA mAb	0.154
		CHH11617 vs MGD785	0.248
		CHH11617 vs CHH995	0.379
		CHH11617 vs MHG1152	0.027
